# Human determinants of age-dependent patterns of death from infection

**DOI:** 10.1016/j.immuni.2024.05.020

**Published:** 2024-07-09

**Authors:** Laurent Abel, Jean-Laurent Casanova

**Affiliations:** 1 Laboratory of Human Genetics of Infectious Diseases, Necker Branch, Inserm U1163, Necker Hospital for Sick Children, Paris, France; 2 Paris Cité University, Imagine Institute, Paris, France; 3 St. Giles Laboratory of Human Genetics of Infectious Diseases, Rockefeller Branch, The Rockefeller University, New York, NY, USA; 4 Department of Pediatrics, Necker Hospital for Sick Children, Paris, France; 5 Howard Hughes Medical Institute, New York, NY, USA

## Abstract

Regardless of microbial virulence (i.e., the global infection-fatality ratio), age generally drives the prevalence of death from infection in unvaccinated humans. Four mortality patterns are recognized: the common U- and L-shaped curves of endemic infections and the unique W- and J-shaped curves of pandemic infections. We suggest that these patterns result from different sets of human genetic and immunological determinants. In this model, it is the interplay between (1) monogenic genotypes affecting immunity to primary infection that preferentially manifest early in life and related genotypes or their phenocopies, including auto-antibodies, which manifest later in life and (2) the occurrence and persistence of adaptive, acquired immunity to primary or cross-reactive infections, which shapes the age-dependent pattern of human deaths from infection.

## INTRODUCTION

There is tremendous interindividual clinical variability in the course of any infection.^1^ No more than a dozen known microbes have the capacity to kill more than 10% of infected individuals, even in the absence of medical prevention and treatment. Fewer than 100 microbes can kill more than 1% of infected individuals. Most of the remaining 3,000 known pathogens are less virulent, triggering life-threatening disease in <1% of infected individuals. The observed infection-fatality ratio is probably the best estimate of microbial virulence in humans. Infection-fatality ratios are, however, not documented as often or as well as case-fatality ratios because B or T cell-dependent infection screens have been performed for only a limited range of infectious agents in the general population. The infection denominator is accurately known only for microbes that are universal commensals. For other microbes, the rare serological screens performed have occasionally revealed surprises in some populations, with microbes that were thought to be highly virulent, such as the causal agent of plague, *Yersinia pestis*, having infected more people than initially thought.^2,3^ Despite the paucity of infection-fatality data, it is commonly thought that infection outcome is driven mostly by qualitative (pathogenicity) and quantitative (inoculum) microbial determinants. However, these determinants account for human interpopulation differences of virulence between microbes better than for human interindividual differences for a given microbe.

The vast, unexplained interindividual clinical variability observed during any infection is known as the “infection enigma.” In other words, what is the root cause of death from infection? This problem, posed at the turn of the 20^th^ century, has received less attention from microbiologists and immunologists than from population and clinical geneticists.^4^ The efforts of these geneticists have progressively given rise to the notion that host determinants—inherited or acquired, overt or covert—pre-exist and underlie life-threatening infectious disease.^1,5^ According to this model of infection, the cause of disease and death lies in the host, whereas the microbe is an environmental trigger that reveals the hitherto silent consequence of this deficiency in the host. Put another way, death from any infection attests to an immunodeficiency, whether inherited or acquired, overt or covert, predisposing the individual to the specific infection concerned. Inherited deficiencies can be rare or common and can confer predispositions to rare or common infections. The study of rare disorders of immunity led to that of common inherited or acquired determinants of infection.^5^ Acquired immunodeficiencies may be driven by external (e.g., immunosuppressants, HIV infection) or internal factors (e.g., autoimmunity against cytokines, “immunosenescence”). The latter process can, paradoxically, be genetically driven. However, disease may not develop in everyone with an inherited or acquired immunodeficiency due to incomplete penetrance. Moreover, immunological abnormalities conferring a predisposition to disease may themselves display incomplete penetrance, not only in patients with inherited genetic lesions but also in patients with an acquired risk factor, potentially due to genetic modifiers in both cases.

A related enigma that has received even less attention relates to the strong dependence of death from infection on age. We do not consider transplacental and sexually transmitted infectious diseases here, the age-dependence of which reflects the specific age at which infection occurs.^6,7^ For almost all other infectious diseases, primary infection typically occurs during childhood, and young children are by far the most vulnerable, with adolescents and young adults being the least susceptible. In older individuals, susceptibility to death generally increases with age, resulting in the classic U-shaped prevalence curve. This is the pattern typical of most endemic infections, whether rare or common, such as tuberculosis. Less frequently, the risk of infectious death may not increase in elderly people, resulting in the classic L-shaped prevalence curve.^8^ This curve is observed for endemic infections as devastating and common as malaria. A first exception to the predominant U and L patterns was provided by the 1918 influenza pandemic, which appeared to follow a unique W-shaped curve, different from the curves observed in more geographically restricted recent influenza pandemics.^9^ People aged 25–34 years were almost as severely affected as children and elderly people, whereas young adults under the age of 25 years and those aged 45–65 years fared better. The coronavirus disease 2019 (COVID-19) pandemic has provided another exception, in the form of a J-shaped curve,^10^ with the risk of death doubling every 5 years of age from childhood onward. There are now many more endemic than pandemic infections, and these two types of infections seem to follow different, non-overlapping patterns, with two patterns specific for each of these groups.

Most, if not all, lethal infections studied epidemiologically and without a specific age-dependent exposure (e.g., transplacental or sexual) follow one of these four patterns: U or L patterns for endemic infections, and W or J patterns for pandemic infections. Why? How do microbial and human determinants account for the different patterns of age-dependent death from infection? This simple but important question has received surprisingly little attention. Here, we attempt to analyze the contribution of known human determinants and to infer plausible and testable models. Indeed, there has been considerable progress in the field of inborn errors of immunity to infection.^1^ Moreover, our knowledge of the human genetic and immunological determinants of infectious diseases has reached an unprecedented level, with 1% and 6.3% of cases of tuberculosis explained by P1104A TYK2 homozygosity and HIV infection,^11–13^ respectively, and 5%, 15%, 20%, and 40% of cases of critical seasonal influenza pneumonia, critical COVID-19 pneumonia, critical Middle East respiratory syndrome (MERS) pneumonia, and West Nile virus encephalitis, respectively, explained by auto-antibodies (auto-Abs) neutralizing type I interferons (IFNs).^14–17^ Many of these determinants strike individuals in specific age groups. This series of discoveries thus provides a unique and timely opportunity to revisit and interpret the epidemiological distributions of death due to endemic and pandemic infections. The four distinctive epidemiologic patterns of prevalence for severe infectious diseases suggest that different mechanisms of immunity to infection operate in children, adults, and the elderly.

## THE OBSERVATIONS: FOUR AGE-DEPENDENT MORTALITY CURVES

**Figure 1.**
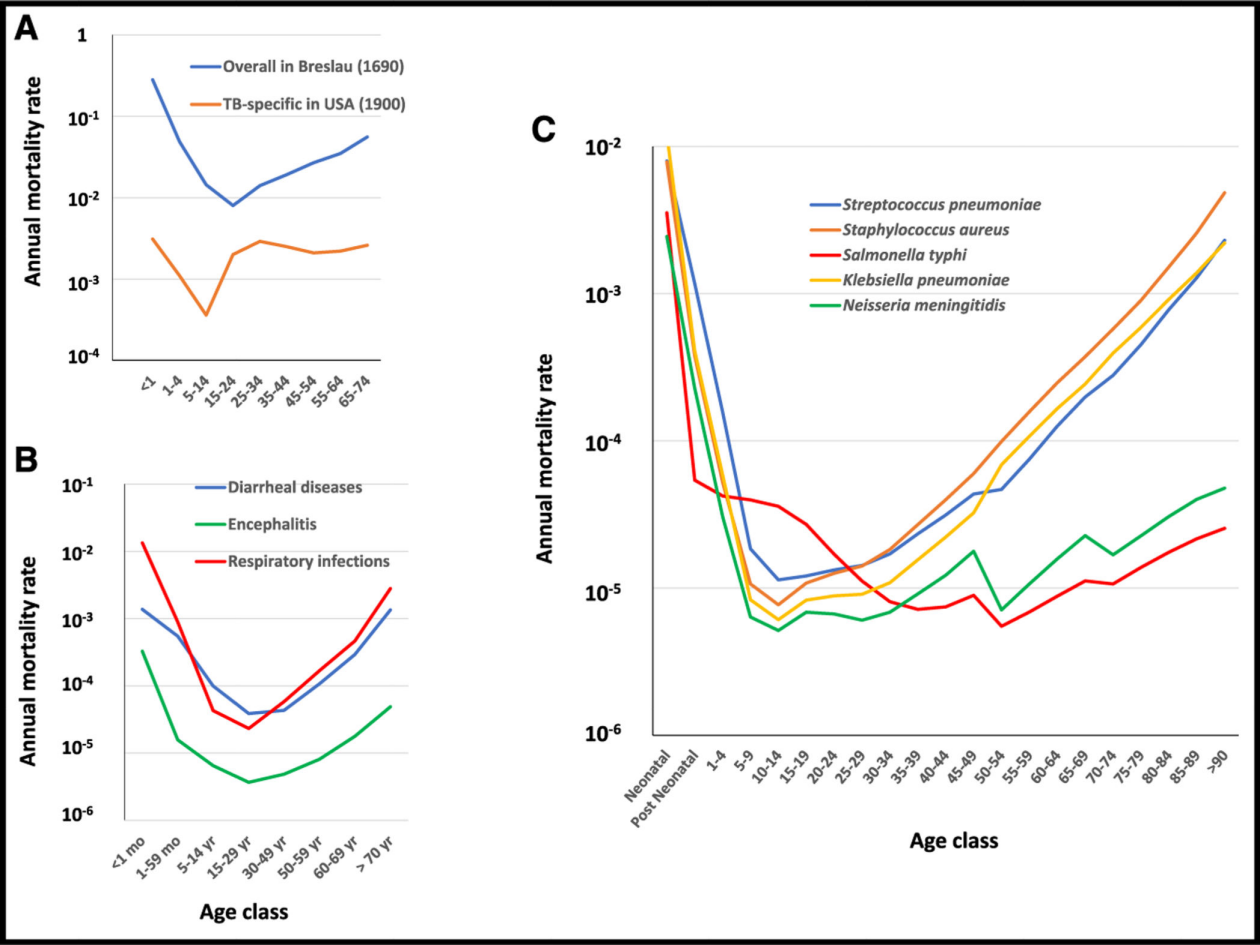
Examples of U-shaped curves (A) Annual mortality rates (log scale) as a function of age before the development of effective preventive measures and treatments for infection: overall mortality observed in Breslau (blue line) by Edmund Halley, who carried out the first reliable life table survey in 1690 (adapted from the book by John Cairns^19^), and tuberculosis-specific rates (orange line) observed in the USA in 1900.^20^ (B) Specific mortality rates for three infectious conditions defined by the WHO in 2019 (https://www.who.int/data/gho/data/themes/mortality-and-global-health-estimates/ghe-leading-causes-of-death): diarrheal diseases (blue line), respiratory infections (red), and encephalitis (green). (C) Specific mortality rates for five of the 14 most lethal bacterial diseases worldwide^24^: *Staphylococcus aureus* (orange), *Streptococcus pneumoniae* (blue), and *Klebsiella pneumoniae* (yellow line) following U-shaped curves, and *Neisseria meningitidis* (green) and *Salmonella typhi* (red) following L-shaped curves. Data for bacterial diseases were obtained from the Institute for Health Metrics and Evaluation (IHME), Seattle, USA (https://vizhub.healthdata.org/gbd-results/). Raw data for Figure 1 are in supplemental Table 1 (data availability).

Historically, infectious diseases were the major killers of human-kind until the beginning of the 20^th^ century and the progressive development of hygiene, vaccines, aseptic surgery, and antimicrobial agents.^1,18^ In the mid-19^th^ century, about 60% of deaths were due to infectious diseases, and their impact at this time can be estimated from overall mortality curves.^8^ In 1690 in Breslau, mortality was very high in the first year of life (28%), strongly decreasing thereafter until early adulthood, and then progressively increasing with age, leading to a classical U-shaped pattern (Figure 1A).^19^ Reliable mortality curves for specific infectious diseases became available from the late 19^th^ century onward. As a classical example, tuberculosis mortality rates in 1900 in the US^20^ follow a similar U-shaped curve (Figure 1A) with a classic “golden age” in adolescents.^21^ Nowadays, life expectancy is much longer, largely due to major improvements in the prevention and treatment of infectious diseases. However, the age-dependent pattern of death from infections for which there are no vaccines has not changed significantly, although the prevalence of such diseases may be lower, thanks to hygiene and antimicrobial drugs. Analysis of the worldwide mortality rates provided by the WHO (https://platform.who.int/mortality) reveals that many infectious conditions, such as “respiratory infections” and “diarrheal diseases” follow a U-shaped curve (Figure 1B). This is also the case for encephalitis, consistent with the U-shaped curve followed by the incidence of herpes simplex virus (HSV)-1 encephalitis.^22,23^ Twelve of the 14 bacterial pathogens with the highest mortality rates worldwide follow U-shaped mortality curves,^24^ including the three most common: *Staphylococcus aureus*, *Streptococcus pneumoniae*, and *Klebsiella pneumoniae* (Figure 1C). Most endemic infections, therefore, kill the very young and the old.

**Figure 2.**
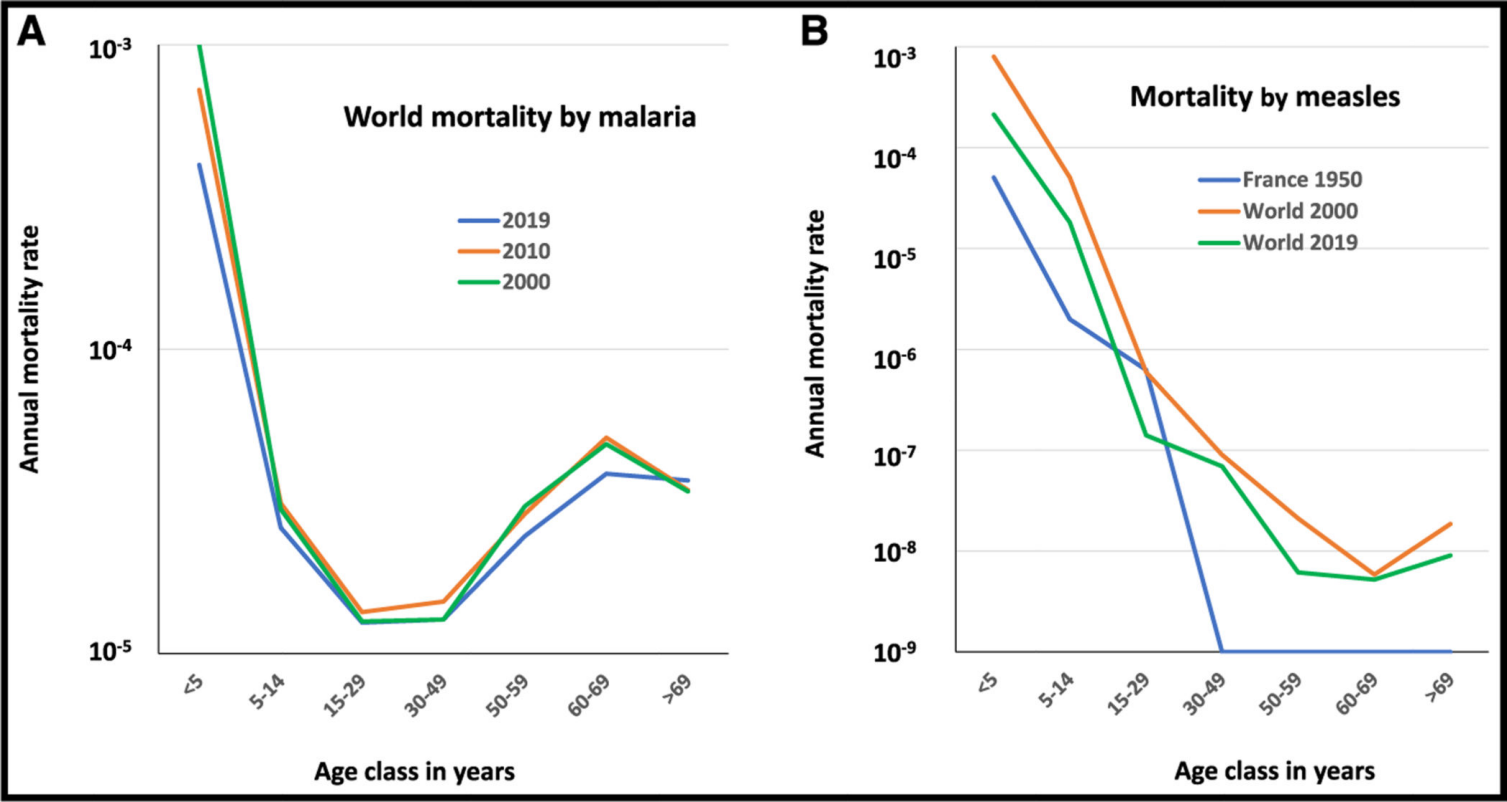
Annual mortality rates (log scale) as a function of age for two infectious diseases with L-shaped curves (A) Worldwide mortality rates for malaria reported in 2000 (green), 2010 (orange), and 2019 (blue) by the WHO. (B) Mortality rates for measles reported by the WHO in France in 1950 before vaccination (blue) and worldwide in 2000 (orange) and 2019 (green). Raw data for Figure 2 are in supplemental Table 2 (data availability).

The U-shaped curve is the most common pattern observed for endemic infectious diseases, but some infections display a different pattern, with high mortality rates in young children but no substantial increase in the risk of death in old age, leading to an L-shaped mortality curve. Based on WHO data, this is the case, for example, for *Plasmodium falciparum* malaria, which displays two orders of magnitude decrease between childhood and adolescence and then a slight increase (about 0.5 orders of magnitude) in subjects over the age of 60 years (Figure 2A). Measles also presents a huge drop of about five orders of magnitude in mortality between early childhood and young adulthood, both in a pre-vaccination context (e.g., in France in 1950) and nowadays worldwide (Figure 2B), with a flat curve after the age of 40 years. Two of the 14 most deadly bacterial pathogens,^24^ *Neisseria meningitidis* and *Salmonella typhi*, have L-shaped mortality curves (Figure 1C). Overall, there is overwhelming epidemiological evidence for a strong age-dependence of mortality rates due to infectious diseases across time and locations. Mortality rates also depend on the preventive and curative measures available, which may vary across countries, but some age-dependent patterns remain constant over space and time, especially for diseases for which there are no effective preventive and therapeutic measures. This is the case, for example, for malaria and for various types of viral encephalitis. In any case, for almost all infectious diseases occurring in endemic conditions with a primary infection in early childhood, young children are by far the most susceptible, and adolescents and young adults are the least susceptible. A higher risk in the elderly is seen for many, but not all, infections. It is worth bearing in mind, however, that life expectancy at birth remained between 20 and 25 years of age until the middle of the 19^th^ century, due to the very high rates of mortality due to infection in children.^18,19^ Moreover, death before reproductive age is reached has a much stronger evolutionary impact.^25,26^ The fundamental feature common to the U and L curves is, therefore, the much greater vulnerability of children than adults to endemic infections.

**Figure 3.**
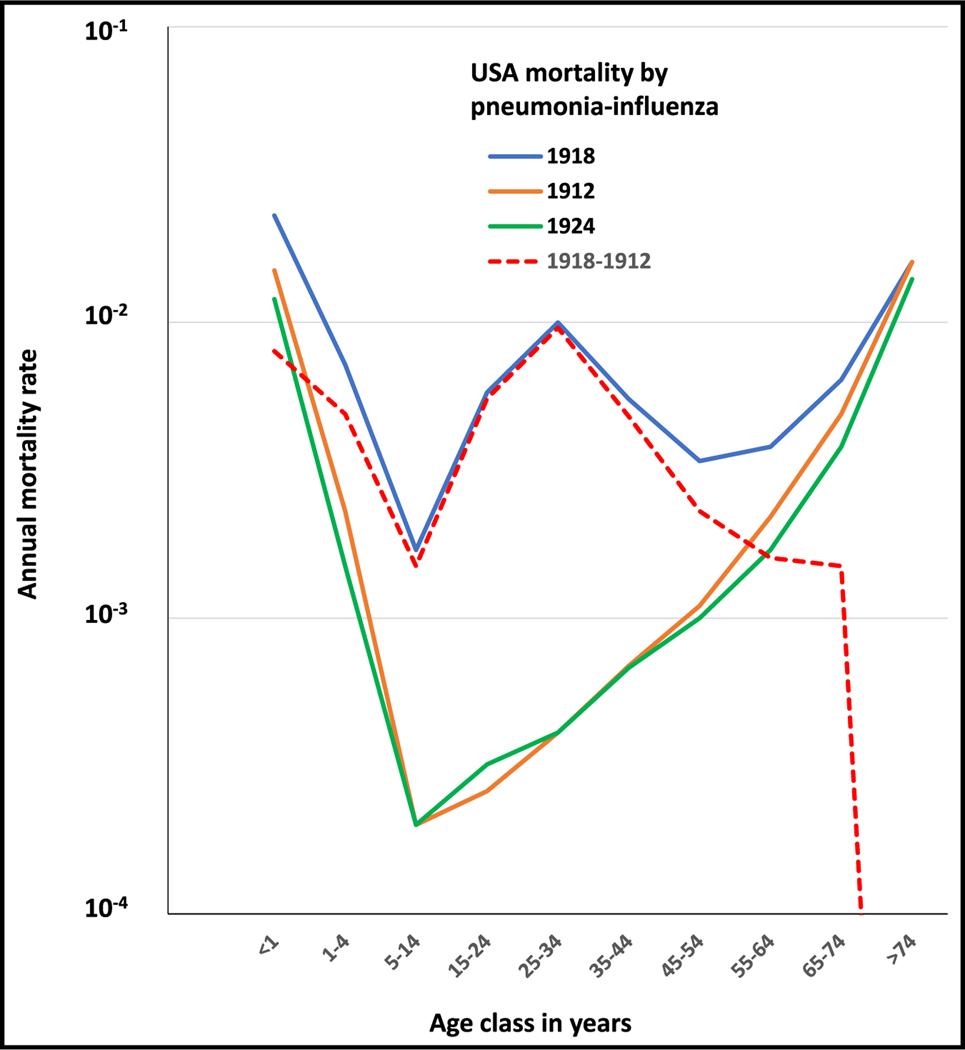
Annual mortality rates (log scale) as a function of age observed in the 1918 influenza pandemic following a W-shaped curve (blue) Figure also displays the mortality rates for seasonal influenza pneumonia 6 years before (1912, orange) and after (1924, green) the 1918 pandemic and excess of mortality for the year of the pandemic (1918) compared with the seasonal flu in 1912 (dotted red line). The data are for the USA and were obtained from Linder and Grove.^20^ Raw data for Figure 3 are in supplemental Table 3 (data availability).

There are only two documented exceptions to the classic U- and L-shaped mortality curves, both observed in sudden, pandemic contexts. The first of these exceptions was provided by the “Spanish” influenza pandemic of 1918–1919, which killed between 2% and 20% of infected individuals, amounting to about 50 million deaths worldwide.^9^ As for other infectious diseases, mortality rates were high in the very young and the elderly. However, the 1918 influenza curve had an unusual specific feature: a high mortality rate in 25- to 34-year-old adults, leading to a unique W-shaped curve. This W-shaped curve was observed with several different sets of data^27–29^ and is shown in Figure 3 for the data from the US.^20^ Interestingly, the curves for deaths from less virulent seasonal influenza in 1912, before the pandemic, and in 1924, after the pandemic, were similar for young children and the elderly but followed the classic U-shaped curve with no particular vulnerability of any intermediate age group (Figure 3). The mortality rate curves for the other two influenza pandemics in 1957 and 1968 were also U-shaped.^30^ To our knowledge, the 1918 influenza pandemic provides the only example of a W-shaped mortality curve. Finally, the excess mortality in 1918 relative to 1912 (dotted line) followed a similar W-shaped pattern except for individuals over the age of 65 years, with a sharp decrease in excess mortality among the elderly. Remarkably, the mortality of individuals over 65 years during the 1918 influenza pandemic was similar to that observed in 1912 for seasonal flu. This was not the case for the pandemics of 1957 and 1968, during which excess mortality was observed in the elderly.^30^

**Figure 4.**
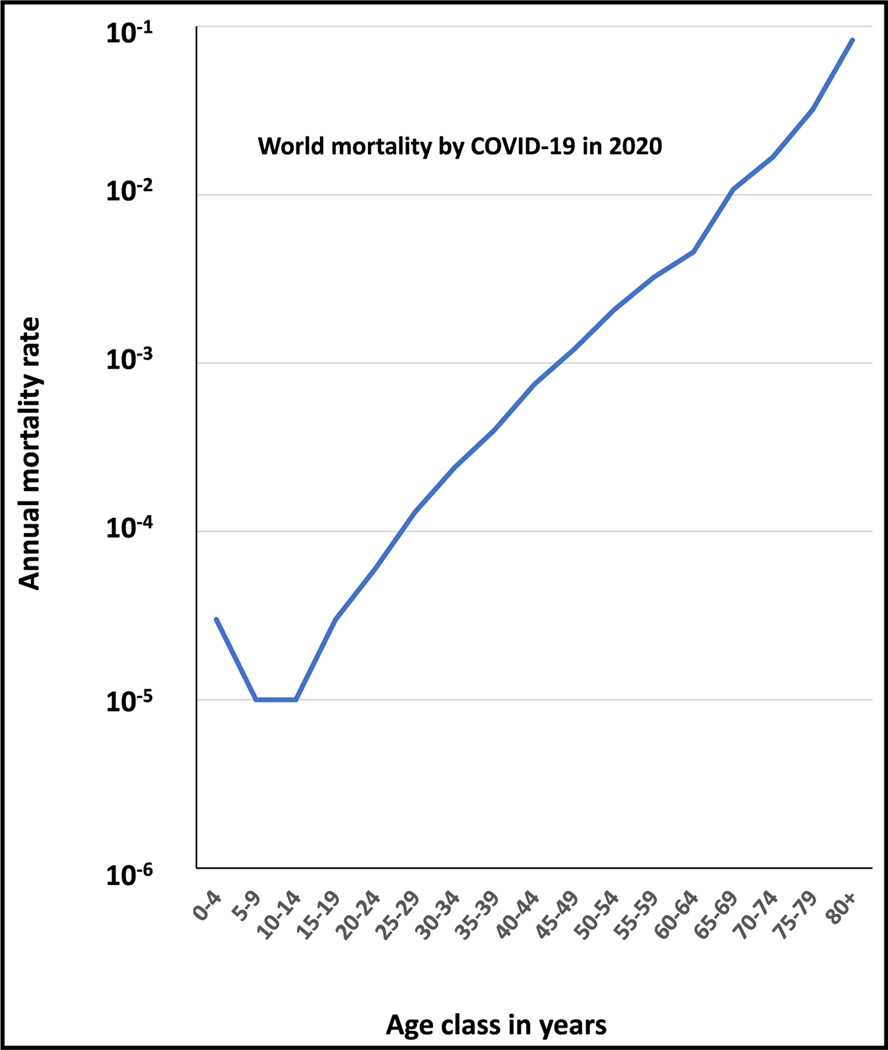
Annual mortality rates (log scale) as a function of age observed in the 2020 COVID-19 pandemic following a J-shaped curve Data are worldwide and were obtained from O’Driscoll et al.^10^ Raw data for Figure 4 are in supplemental Table 4 (data availability).

Another remarkable exception to the U and L curves was observed with the recent COVID-19 pandemic, which killed at least 7 million of the estimated 700 million infected individuals. In the absence of medical diagnosis, prevention, and care, global mortality would probably have been similar to that for the 1918 influenza pandemic. Molecular diagnosis was available at the very beginning of the pandemic, whereas no means of virological diagnosis were available in 1918. Moreover, an effective vaccine became available about a year into the pandemic. More relevant to our discussion, about 5%–10% of unvaccinated infected individuals had hypoxemic COVID-19 pneumonia. These patients benefited from oxygen therapy and other forms of intensive care, resulting in an overall mortality rate of between 0.5% and 1%. The global virulence of the 1918 influenza virus and the 2019 severe acute respiratory syndrome coronavirus 2 (SARS-CoV-2) are, therefore, similar, but these two pandemics presented completely different patterns of age-dependent mortality. The estimated mortality curves for COVID-19 estimated in 2020, before the vaccine became available, followed a clear J-shaped pattern (Figure 4).^10^ Mortality rates were the lowest in children between 5–14 years of age (about 0.001%), subsequently increasing exponentially with age (approximately doubling with every 5 years of age, the largest increase being a 2.6-fold increase between the 75–79 years age group and the >80 age group) and reaching about 10% in subjects older than 80 years. These mortality rates were shown to be consistent across different settings and can therefore be considered robust estimates for the population infected with SARS-CoV-2.^10^ The contrast between the oldest studied pandemic (1918) and the most recent one (2020), about a century later, both caused by ssRNA respiratory viruses (one a positive-strand and the other a negative-strand virus, admittedly) and with a similar global virulence, is particularly striking.

## THE INTERPRETATIONS: FOUR MECHANISMS OF DISEASE

There is growing evidence to suggest that monogenic inborn errors of immunity contribute to the high mortality of infectious diseases in children.^1,5,18,31^ The classic “primary immunodeficiencies” are rare Mendelian traits that underlie multiple infectious diseases. Other rare monogenic defects confer a predisposition to specific infections, typically with incomplete penetrance (non-Mendelian). Death from infection before 6 months of age is probably due to the most severe inborn errors, which cannot be compensated with specific maternal Abs, but a broader range of disorders can be lethal in older children. Most of such inborn errors probably preferentially impair innate (leukocytic) and/or intrinsic (non-leukocytic) immunity, with fewer having a selective impact on adaptive immunity. As proposed in 2010, death from infection in childhood is unlikely to be polygenic, or at least due to common variants that underlie a dominant trait.^8^ Perhaps the best example to date is provided by the finding that 5%–10% of cases of childhood HSV-1 encephalitis are due to single-gene inborn errors of brain immunity, which is often type I IFN-dependent.^32–34^ Malnutrition may also increase the likelihood of death from infection during childhood.^35^ The second defining feature of the U-shaped curve is the increase in mortality rates in the elderly. We previously suggested that age-dependent somatic and epigenetic factors, together with polygenic predisposition, may progressively impair immunity.^8^ We did not initially suspect that death from infection might be due to common variants underlying a recessive trait, as illustrated by homozygosity for P1104A TYK2 underlying 1% of European cases of tuberculosis.^11,12^

We then discovered another hitherto unsuspected major factor: auto-Abs that neutralize type I IFNs, which we referred to as phenocopies of inborn errors of type I IFN immunity.^36^ Indeed, such auto-Abs underlie viral infections seen in patients with inborn errors that impair the production of or the response to type I IFNs. However, these auto-Abs can themselves be due to inborn errors of tolerance to type I IFNs.^37–39^ Rigorously, they should probably not be considered as phenocopies, which are literally due to environmental impacts, as opposed to causal genotypes.^40,41^ The same viral infections can be caused by inborn errors of type I IFNs (production of or responses to type I IFNs) or inborn errors of tolerance to type I IFNs (underlying auto-Abs against them). With a prevalence of about 0.5%–1% before 65 years of age, these auto-Abs become increasingly common after the age of 70 years, reaching a prevalence of 4%–8%.^14,15^ They underlie 5%, 15%, 20%, and 40% of cases of critical seasonal influenza pneumonia, critical COVID-19 pneumonia, critical MERS pneumonia, and West Nile virus encephalitis (WNVE), respectively.^14–17^ Other endemic infections with prevalence rates increasing in the elderly, such as herpes zoster,^42^ may also fall into this category due to auto-Abs against specific cytokines or other molecules involved in host defense. It is tempting to speculate that the occurrence of auto-Abs against IFN-γ might also underlie the reactivation of *M. tuberculosis* from latency and the development of tuberculosis in the elderly. Comorbid conditions in the elderly may provide an alternative or additional explanation. Inherited and acquired immunodeficiencies together account largely for endemic U-shaped mortality curves for infection, with high mortality rates at the extreme ends of the age range.

The high mortality rate in children observed for diseases with an L curve may also reflect the contribution of inborn errors of immunity. Admittedly, for most of these conditions, including malaria, inborn errors have not yet been reported, possibly reflecting a lack of ad hoc studies. For measles, there are too few outbreaks to draw any firm conclusions, given the wide vaccination coverage. However, it is perhaps not coincidental that measles outbreaks with high mortality rates have been reported in countries with poor vaccination rates and a high prevalence of loss-of-function *IFNAR1* and *IFNAR2* alleles, in Western Polynesia and the Arctic region, for example.^43,44^ Moreover, there are inborn errors that protect against malaria, as exemplified by homozygosity for a common DARC allele that prevents Duffy antigen expression on erythrocytes, rendering the individuals concerned resistant to infection with *P. vivax*.^45^ Heterozygosity for HbAS also provides 10-fold protection against severe forms of *P. falciparum* malaria.^4^ The contribution of other common variants to severe malaria remains modest, relative to the HbAS effect.^46^ Importantly, rare variants have not been studied in detail. The decrease in mortality after the age of 5 years, resulting in a subsequently flat curve, suggests that acquired immunity is solid and persists with age, perhaps due to frequent reinfections in endemic conditions. It also suggests that no autoimmunity develops against key molecules involved in defense against these microbes, or, more generally, that there is no age-dependent phenocopy of the inborn errors underlying childhood death, or at least that these phenocopies do not overcome adaptive immunity against these pathogens.

Overall, the L curve attests to the efficacy of adaptive immunity driven by T and/or B cells specific for the endemic microbe. This is neatly illustrated by the incomplete penetrance of autosomal recessive Toll/interleukin-1 receptor domain-containing adaptor protein (TIRAP) and dominant OTULIN deficiencies, which underlie life-threatening staphylococcal disease until Abs against staphylococcal antigens are able to overcome the genetic deficiency and protect the patients.^47,48^ Another illustration is provided by the massive improvement in clinical outcome after the age of 10 years in patients with autosomal recessive IRAK4 or MyD88 deficiency.^49^ Streptococcal B disease in neonates provides an extreme example, as this condition is almost never seen in older individuals.^50^ Adaptive immunity to the same or related pathogens can, therefore, mitigate the impact of inborn errors on infections with endemic pathogens. Moreover, most individuals with these inborn errors suffer from life-threatening disease during primary infection in childhood. Both phenomena may account for the L-shaped curve.

The W- and U-shaped curves have high mortality rates in children and the elderly in common. The same explanations for both curves can account for this high mortality at both ends of the age spectrum: inborn errors of immunity for children and their acquired phenocopies for the elderly. Both inborn errors of type I IFN and auto-Abs neutralizing type I IFNs can underlie severe seasonal influenza, with inborn errors preferentially affecting children and auto-Abs most frequently found in the elderly.^17,51–53^ There is no reason to suspect that the same disorders would not underlie severe cases of pandemic influenza. Milder disorders of type I IFN, whether inherited or acquired, may even underlie severe disease caused by more virulent influenza viruses. Indeed, heterozygosity for rare variants of the type I IFN-inducible gene *MX1* variants that do not seem to underlie death from seasonal flu can confer a predisposition to avian flu.^54^

Nevertheless, the main specificity of the “W” curve is the high mortality rate observed in young adults aged 25 to 34 years. This pattern was specific to the 1918 influenza pandemic and was not observed during the 1957 and 1968 pandemics.^30^ Several hypotheses were put forward to explain this feature. The most plausible explanation is that individuals over the age of 30 years in 1918 had some protective immunity due to prior exposure to a related influenza strain.^27^ Indeed, there is indirect evidence that H3-like viruses started to circulate around 1889, whereas the viruses circulating before that were of the H1 subtype. The observation of an absence of excess mortality in the elderly during the 1918 pandemic, contrasting with seasonal flu (e.g., in 1912), is also consistent with this hypothesis. Elderly individuals in 1918 may also have been protected by prior exposure to influenza viruses of the H1 subtype. The W curve therefore provides an “experimental” validation of the mechanisms underlying the L and U curves, with adaptive immunity to the same or related pathogens progressively buffering the deleterious impact of inborn errors of innate or intrinsic immunity, and their absence accounting for a rebound of death risk in a particular age class.

Inborn errors of TLR3- and TLR7-dependent type I IFN immunity underlie critical COVID-19 pneumonia, especially in individuals under 60 years old.^55–57^ They were also found in young children, especially recessive forms,^58^ potentially accounting for the slightly higher mortality observed in children under the age of 5 years. Inborn errors of type I IFN immunity, particularly the most severe recessive disorders, may have been revealed by endemic viral infections before the 2020 COVID-19 pandemic, accounting for their decrease in frequency with age, especially after 5 years of age. During the COVID-19 pandemic, it was discovered that auto-Abs neutralizing type I IFNs could account for about 20% of deaths in adults aged 20 to 99 years.^14^ The sharp increase in mortality in individuals over the age of 65 years can be explained largely by the increase in the frequency of auto-Abs against type I IFNs observed in the general population in this age group. By contrast, the frequency of these auto-Abs remains stable between the ages of 20 and 65 years. They are also present in children, at a frequency similar to that found in young adults, in whom they also strongly increase the risk of COVID-19 pneumonia.^59^

One intriguing question that remains unanswered concerns the steady increase in COVID-19 mortality between the ages of 15 and 60 years. The lack of a dip at intermediate ages, as in the W curve, and the log-linear nature of the curve obtained suggest that previous endemic and pandemic coronavirus infections had no significant impact. However, partial protection due to infection with a related endemic coronavirus strain might decrease with age. Alternatively, low levels of auto-Abs against type I IFNs, increasing in frequency with age and undetected *in vitro*, may have an effect. As auto-Abs account for only 20% of cases, there are probably other age-dependent phenocopies playing a role in the elderly. There may be an age-dependent decline of type I IFN levels in the respiratory tract (independently of the viral challenges stimulating the production of this cytokine).^60–62^ Somatic mutations, which increase with age, may also play a role.^63^ Nevertheless, the J curve and the mechanisms at work are not specific to COVID-19. Indeed, 40% of WNVE cases are caused by auto-Abs neutralizing type I IFNs.^16^ As for COVID-19, the incidence of WNVE increases steadily after the age of 50 years old, the risk being highest in individuals over the age of 70 years (defining a “flatter” version of the J curve).^22,64^ Overall, in the presence of a new infectious challenge for which there is no pre-existing immunity, an increase in risk with age due to auto-Abs neutralizing a key component of host defense may account to a large extent for the J-shaped curve.

## FUTURE DIRECTIONS

One characteristic common to the four curves of mortality due to infection is that mortality rates are lowest in the 5–15 years age group, a period that Dubos called the golden age with reference to tuberculosis^21^ and that Ahmed et al.^27^ referred to as the “honeymoon period.” This feature is common not only to the U, L, and W curves but also to the J curve. The vast majority of primary infections occur in young children before the age of 5 years, and this is probably a key determinant of the higher mortality of this age group. In highly endemic settings, in which primary infection occurs principally before the age of 5 years, inborn errors of immunity to infection are detected mostly in the members of this age group (the U and L curves). For pandemic infections, inborn errors of immunity specific to the new pathogen may be revealed at all ages, although some might also have been revealed earlier by endemic infections due to related microbes. Additional hypotheses for this honeymoon period were discussed by Ahmed et al.,^27^ including a greater vigor of leukocytes at their chronological peak, after their maturation in infancy, and before their decline during adulthood.

Another feature revealed by the curves is that elderly people are vulnerable to most infections. For both endemic and pandemic infections, phenocopies of rare inborn errors of immunity observed childhood should be considered. Autoimmunity against components of host defense, which occur preferentially in the elderly, may attest to a trade-off with adaptive immunity, which has been mainly selected evolutionarily for host defense prior to and during the age of reproduction.^26^ Some cytokine auto-Abs may be rare (auto-Abs against IL6, IL17, granulocyte macrophage colony-stimulating factor [GM-CSF], and type II IFN), but others are common in the general population (type I IFN). Moreover, auto-Abs against type I IFNs can underlie various viral illnesses of the elderly, including COVID-19, influenza, HSV-1 encephalitis, and WNVE. Importantly, these autoimmune phenocopies may themselves be genetically driven, including by single-gene inborn errors of immunity.^38^ Inborn errors due to common variants can also be involved, as illustrated by homozygosity for P1104A TYK2, underlying about 1% of cases of tuberculosis in adult Europeans. These are two human determinants of infectious death that we did not consider in 2010.^8^ There may be other types of genetic lesions that underlie infections in the elderly, including digenic, oligogenic, and polygenic predispositions, or somatic mutations that also increase in frequency with age.^63^ An epigenetically driven decline of adaptive immunity is also possible.^65,66^ A decline of innate (leukocytic) and intrinsic (non-leukocytic) immunity with age may also be involved, as recently illustrated by the increased susceptibility with age of nasal epithelial cells to SARS-CoV-2 infection, in relation to impaired type I IFN production in the elderly.^60–62^

## CONCLUDING REMARKS

**Figure 5.**
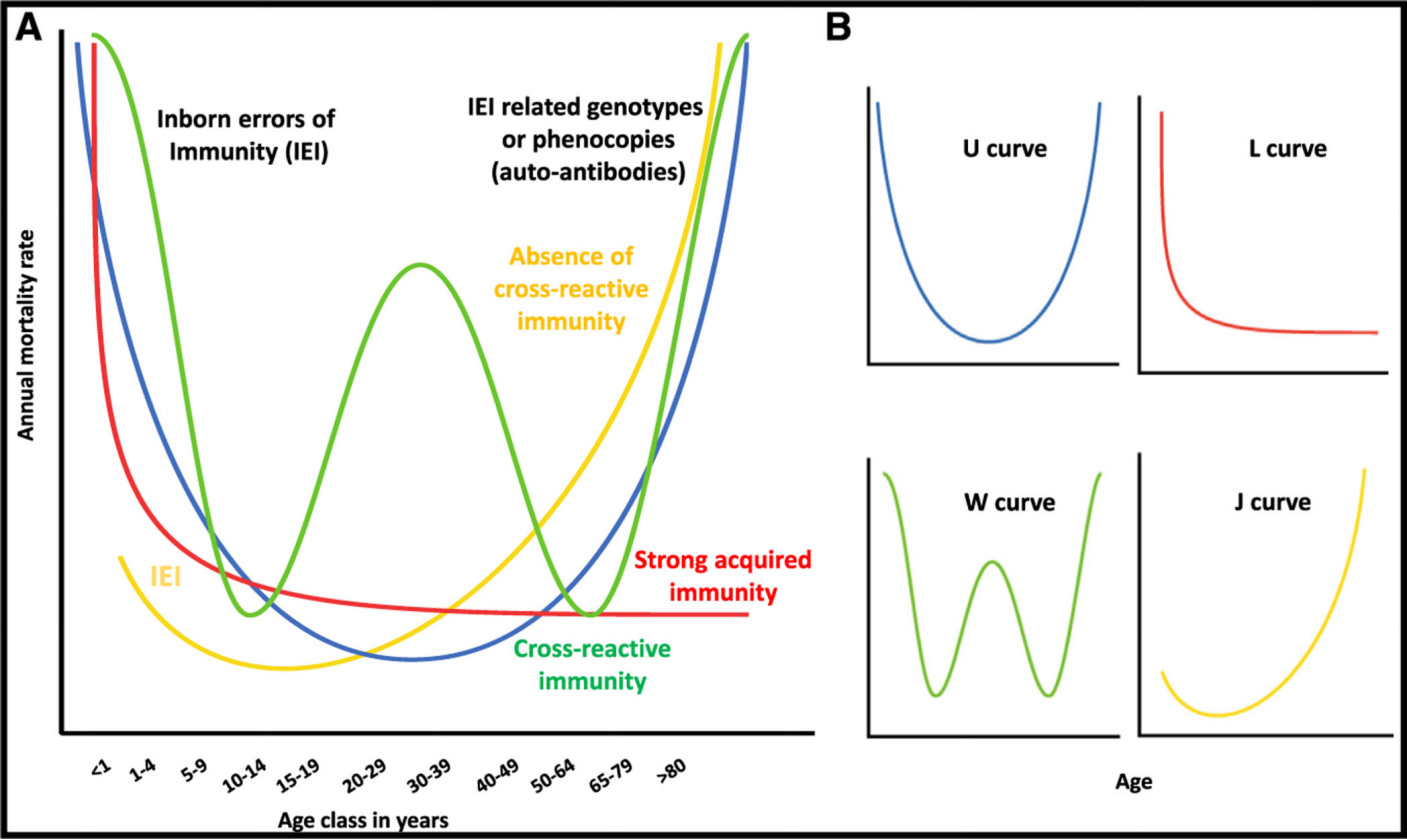
Schematic representation of the four age-dependent patterns of death from infection (A) Hypothetical determinants specific to a given pattern are shown in the same color as the corresponding curve, whereas those common to several patterns are shown in black. (B) Separately provides the four age-dependent patterns.

The four curves of mortality from infection have long been known (U, L, W) or were recently (J) described. However, they have never been explained, either causally and mechanistically or hypothetically or speculatively. We propose here hypothetical causes and mechanisms of disease that could explain these four curves (Figure 5). The highly variable vulnerability of individuals between 15 and 60 years of age remains poorly explained. The reasons for the observed susceptibility at the extreme ends of the human lifespan are beginning to be understood, but the factors driving susceptibility in intermediate age groups remain unknown. Research in this field would certainly benefit from other perspectives, which propose alternative or additional hypotheses. As causes and mechanisms have not been sought for most infections, and the cause and mechanism of disease remain unexplained even for the vast majority of patients for the few infections that have been studied, we recognize that our proposed model is largely hypothetical. Nevertheless, it is not contradicted by any existing dataset. It is even consistent with all existing data, including inborn errors of immunity and their autoimmune phenocopies.^5^ The main objective of this essay is, therefore, to verbalize plausible hypotheses, which can and should be observationally and experimentally tested. Genetic and immunological studies of large cohorts of patients with a given infectious disease are warranted, including studies based on whole-genome sequencing and the detection of auto-Abs.

## Supplementary Material

Supp.Tables

## Data Availability

“Supplemental_tables_Abel_Casanova” are available using Mendeley Data, V1, https://doi.org/10.17632/t24h6pchmv.1.
